# PPARγ agonists delay age‐associated metabolic disease and extend longevity

**DOI:** 10.1111/acel.13267

**Published:** 2020-11-21

**Authors:** Lingyan Xu, Xinran Ma, Narendra Verma, Luce Perie, Jay Pendse, Sama Shamloo, Anne Marie Josephson, Dongmei Wang, Jin Qiu, Mingwei Guo, Xiaodan Ping, Michele Allen, Audrey Noguchi, Danielle Springer, Fei Shen, Caizhi Liu, Shiwei Zhang, Lingyu Li, Jin Li, Junjie Xiao, Jian Lu, Zhenyu Du, Jian Luo, Jose O. Aleman, Philipp Leucht, Elisabetta Mueller

**Affiliations:** ^1^ Division of Endocrinology Diabetes and Metabolism NYU Grossman School of Medicine New York NY USA; ^2^ Shanghai Key Laboratory of Regulatory Biology Institute of Biomedical Sciences and School of Life Sciences East China Normal University Shanghai China; ^3^ Division of Endocrinology, Diabetes and Metabolism NYU Grossman School of Medicine New York NY USA; ^4^ Medical Service Veterans Affairs New York Harbor Healthcare System New York NY USA; ^5^ Department of Orthopedic Surgery NYU Grossman School of Medicine New York NY USA; ^6^ Murine Phenotyping Core facility NHLBI National Institutes of Health Bethesda MD USA; ^7^ School of Physical Education & Health Care East China Normal University Shanghai China; ^8^ LANEH School of Life Sciences East China Normal University Shanghai China; ^9^ Cardiac Regeneration and Ageing Lab Institute of Cardiovascular Sciences School of Life Science Shanghai University Shanghai China

**Keywords:** aging, adipose tissue, metabolism, rosiglitazone, PPARγ

## Abstract

Aging leads to a number of disorders caused by cellular senescence, tissue damage, and organ dysfunction. It has been reported that anti‐inflammatory and insulin‐sensitizing compounds delay, or reverse, the aging process and prevent metabolic disorders, neurodegenerative disease, and muscle atrophy, improving healthspan and extending lifespan. Here we investigated the effects of PPARγ agonists in preventing aging and increasing longevity, given their known properties in lowering inflammation and decreasing glycemia. Our molecular and physiological studies show that long‐term treatment of mice at 14 months of age with low doses of the PPARγ ligand rosiglitazone (Rosi) improved glucose metabolism and mitochondrial functionality. These effects were associated with decreased inflammation and reduced tissue atrophy, improved cognitive function, and diminished anxiety‐ and depression‐like conditions, without any adverse effects on cardiac and skeletal functionality. Furthermore, Rosi treatment of mice started when they were 14 months old was associated with lifespan extension. A retrospective analysis of the effects of the PPARγ agonist pioglitazone (Pio) on longevity showed decreased mortality in patients receiving Pio compared to those receiving a PPARγ‐independent insulin secretagogue glimepiride. Taken together, these data suggest the possibility of using PPARγ agonists to promote healthy aging and extend lifespan.

## Graphical abstract

In this manuscript, we show that chronic use of low doses of antidiabetic drugs that target the nuclear receptor PPARgamma preserves metabolic organ function and cognitive abilities and decreases depression‐ and anxiety‐like symptoms. Through functional, histological, and molecular analyses, we demonstrated that treated mice have improved adipose tissue homeostasis, reduced sarcopenia, decreased inflammation, and improved mitochondrial functionality. In addition, our longevity studies demonstrated lifespan extension in mice and increased survival in humans in response to TZD. These data support the possible long‐term use of low doses PPARgamma ligands to counteract aging.

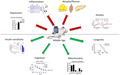

## 1 INTRODUCTION

Aging is a major risk factor for various disorders affecting multiple organs (Dillin et al., 2014). A number of studies have provided evidence to support the value of a balanced diet and physical activity to delay or prevent age‐related health problems (Warner et al., 2005). Interventions such as caloric restriction, the Mediterranean diet, and moderate levels of physical exercise have been suggested to be effective in attenuating aging‐related pathologies and in improving metabolic dysfunction and neurodegenerative disease (Gremeaux et al., 2012; Guarente & Picard, 2005), and have been considered as potential strategies to extend lifespan in human subjects. In addition to behavioral interventions, a number of pharmacological treatments leading to either suppression of chronic inflammation, improvement of insulin sensitivity via the use of antidiabetic compounds (Harrison et al., 2019), or through increased mitochondrial activity have been reported to be beneficial in counteracting age‐associated diseases and to extend lifespan (Carrico et al., 2018).

PPARγ is a critical regulator of adipocyte biology and lipid metabolism, shown to be involved in the control of differentiation, lipid storage, and brown remodeling of white fat (Mueller et al., 2002; Ohno et al., 2012; Tontonoz & Spiegelman, 2008). In addition, PPARγ regulates glucose homeostasis and has anti‐inflammatory and anti‐cancer effects (Choi et al., 2010; Mueller et al., 2000). We have recently provided evidence demonstrating that PPARγ deficiency in subcutaneous fat of aging mice exacerbates age‐associated obesity and metabolic decline (Xu et al., 2018). Further support for PPARγ specific role in aging is provided by studies of mice either hypomorphic for Pparγ or Pparγ2‐deficient demonstrating that modulation of PPARγ levels impacts lifespan (Argmann et al., 2009). These data strongly suggest the potential importance of PPARγ in the regulation of metabolic dysfunction in aging.

It has been shown that thiazolidinediones, such as rosiglitazone (Rosi) and pioglitazone (Pio), are specific PPARγ full agonists acting as insulin sensitizers (Mohanty et al., 2004). These compounds were approved by the Food and Drug Administration for the treatment of diabetes. In addition, they have been reported to be beneficial in counteracting inflammation (Mohanty et al., 2004) and to have a neuroprotective effect, both in animal models and in humans (Moutinho & Landreth, 2017).

Given that PPARγ ligands exert an anti‐inflammatory effect and have insulin‐sensitizing properties, we assessed their potential benefit as anti‐aging agents. Here we report that long‐term treatment of aging mice with Rosi improves insulin sensitivity, prevents tissue atrophy, relieves anxiety‐ and depression‐like symptoms, and improves cognitive function. Our molecular analysis revealed that Rosi treatment decreases the expression of inflammatory gene programs and improves mitochondrial functionality in multiple metabolic organs. Furthermore, we showed that low dose, chronic Rosi treatment started in 14‐month‐old mice extended lifespan. Based on these preclinical data, we conducted a retrospective analysis of the effects of Pio in human subjects and demonstrated that patients receiving Pio live longer than those on glimepiride, an insulin secretagogue belonging to the sulfonylurea class. Overall, the results of the present study suggest that PPARγ ligands improve healthspan and exhibit a life‐extending role not previously appreciated.

## 2 RESULTS

### 2.1 Low doses of rosiglitazone improve insulin sensitivity and prevent adipose tissue loss in aging mice

**FIGURE 1:**
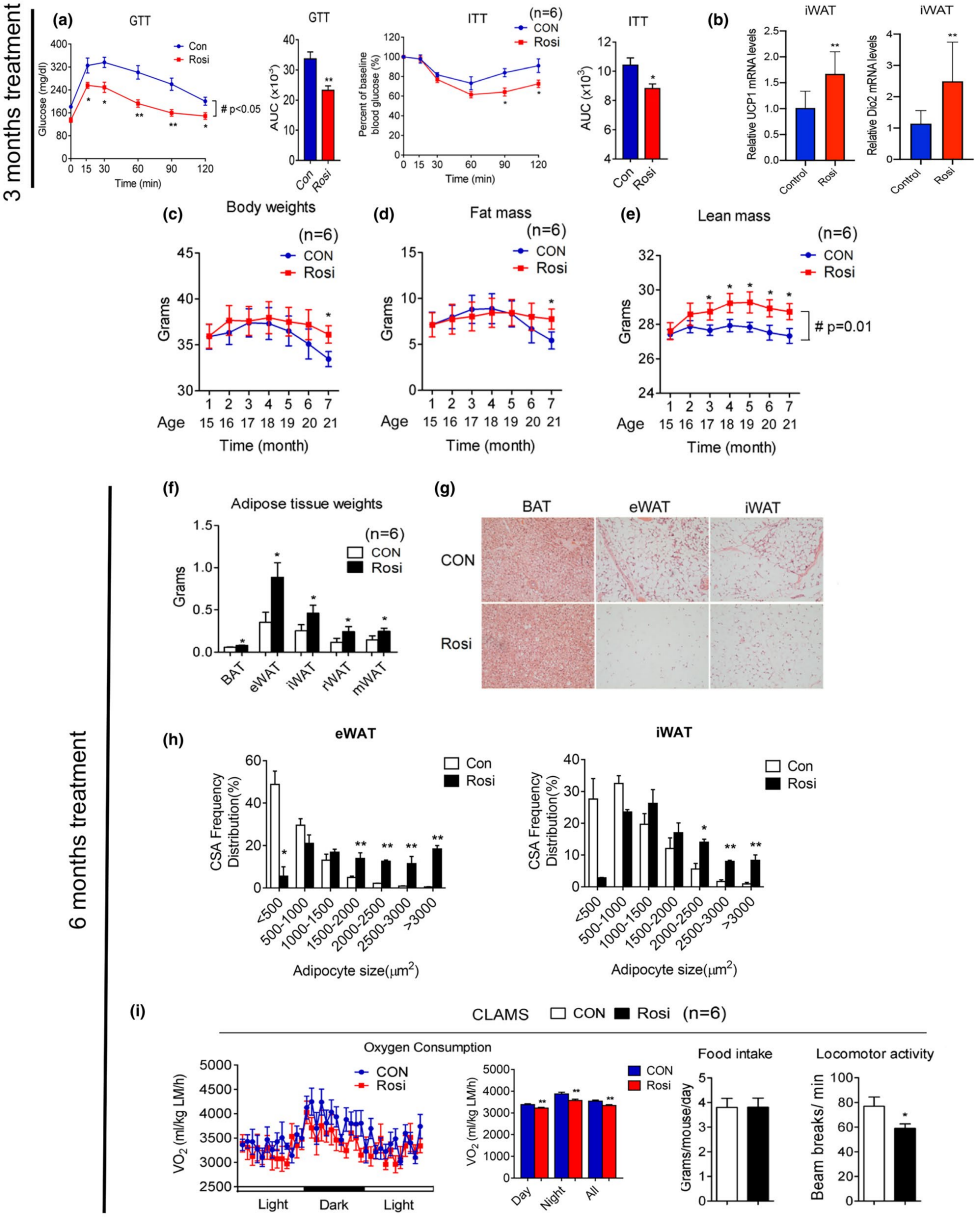
Rosiglitazone treatment in aging mice improves insulin sensitivity and prevents age‐associated adipose tissue loss. (a–i) Analysis of aging mice treated with or without 1 mg/kg rosiglitazone. (a) Insulin sensitivity of mice at 17 months of age, after 3 months treatment, as shown by glucose and insulin tolerance test and quantification of area under the curve (AUC) (n = 6); (b) UCP1 and Dio2 levels in control and treated 17‐month‐old mice, after 3 months of treatment in iWAT (n = 10); (c–e) body weight, fat mass, and lean mass of 14‐month‐old mice over treatment period (n = 6). (f) Adipose tissue weight and (g) representative H&E images of brown fat (BAT), epididymal fat (eWAT), and subcutaneous fat (iWAT) and (h) quantification of adipocyte size after 6 months of treatment of 14‐month‐old mice. (i) Oxygen consumption values obtained via CLAMS analysis, normalized to lean mass, and average food intake and locomotor activity in mice at 20 months of age, after 6 months of either control or Rosi‐containing diet. Data are presented as mean ± SEM. Student t tests were performed to compare control‐ and Rosi‐treated groups (*p < 0.05, **p < 0.01). Repeated measures ANOVA were performed for a, c–e to compare the differences over time (#*p* < 0.05)

To determine the effects of Rosi treatment started in 14‐month‐old mice, we used an oral dose of Rosi of 1 mg/per kg of weight/per day, equivalent to 1/10–100 the amount previously used to treat diabetes (Feng et al., 2011), prevent tumorigenesis (Cellai et al., 2010), and stimulate browning of white fat in animal models (Ohno et al., 2012). Given the known role of Rosi as an insulin sensitizer, we first performed glucose and insulin tolerance tests to confirm Rosi's effectiveness at the low doses used in regulating glucose metabolism in 14‐month‐old mice. As shown in Figure 1a and in Figure S1a, 17‐month‐old mice, at the end of their 3 months treatment period with Rosi, showed improved insulin sensitivity compared to controls. Further analysis in isolated cells obtained from tissues of these control and treated mice demonstrated that Rosi treatment was associated with increased glucose uptake in adipocytes, in particular in those derived from epididymal fat, and in myocytes (Figure S1b).

Furthermore, analysis of UCP1 and Dio2 gene expression revealed that iWAT underwent molecular changes consistent with increased browning (Figure 1b). These data suggest that low doses of Rosi in 14‐month‐old mice can effectively improve insulin sensitivity, one of the hallmarks of metabolic aging (Ma et al., 2014), and are sufficient to stimulate browning of fat tissues.

It has been previously shown that, during aging, adipose tissues undergo increased inflammation and progressive expansion followed by adipopenia and fibrosis. We therefore monitored weight and body mass over time and analyzed fat tissue characteristics of control and treated mice at the end of 6 months of treatment to determine whether chronic exposure to Rosi in 14‐month‐old mice would affect these age‐associated metabolic changes. As shown in Figure 1c,d, as expected (Miller & Wolfe, 2008), control mice displayed progressive loss of body weight and fat mass. In contrast, mice treated with Rosi remained stable throughout the treatment period (Figure 1c–e). The absence of loss of adipose tissue in Rosi‐treated mice was reflected in the increased weight of fat depots, which was accompanied with increased adipocyte size (Figure 1f–h). In addition, Rosi‐treated mice showed increased weight in tissues such as liver, muscle, and pancreas and triglycerides accumulation in liver (Figure S2) Analysis of energy expenditure in control and treated mice revealed reduced oxygen consumption and locomotor activity in Rosi‐treated mice, with no changes in food intake (Figure 1i and Figure S3a). Respiratory quotient (RQ) analysis showed a shift in substrate utilization in mice treated with Rosi during the night (Figure S3b). Consistently with the VO2 results, energy expenditure (EE) data calculated per mouse did not show a difference between groups, while EE normalized to lean mass showed significant decreases in the values during the day, night, and all‐day period in the Rosi‐treated mice (Figure S4). These data demonstrate that chronic treatment of 14‐month‐old mice with low doses of Rosi prevents age‐dependent adipose tissue loss and contributes to maintenance of adipose tissue homeostasis.

### 2.2 Rosiglitazone reduces inflammation, fibrosis, and atrophy in aging tissues

**FIGURE 2:**
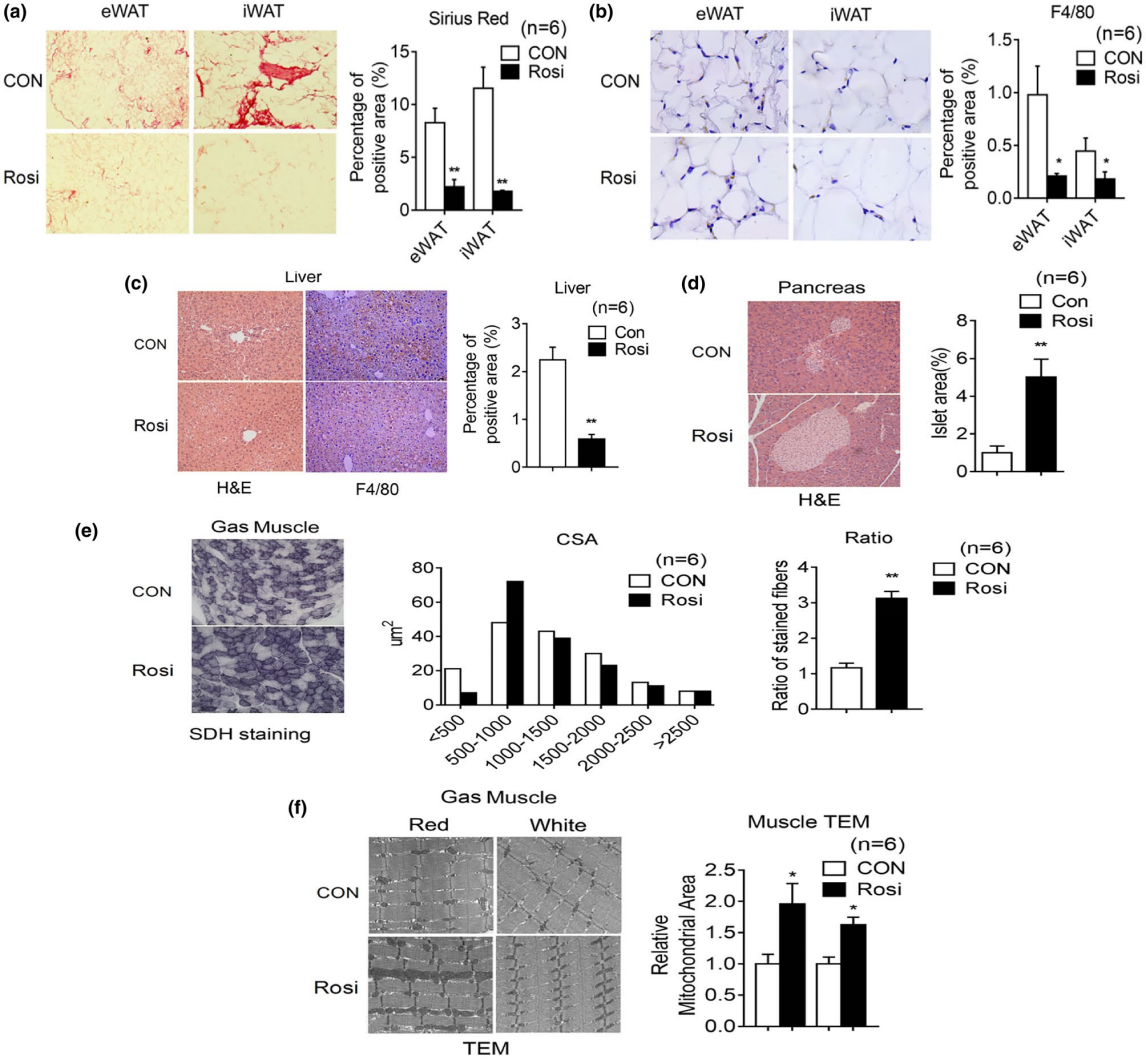
Rosiglitazone treatment in aging mice reduces inflammation, increases oxidative fiber number, and mitochondrial area in muscle. (a–i) Analysis of 20‐month‐old mice after 6 months of treatment, either with control diet or diet supplemented with Rosi (1 mg/kg of mouse weight/day) (n = 6). Representative image of Sirius Red staining (a) and F4/80 staining (b) of eWAT and iWAT with quantification. (c) Representative H&E images and F4/80 staining of liver with quantification. (d) Representative H&E images of pancreas and quantification of islet area. (e) Cross‐sectional area (CSA) of fiber of gastrocnemius (Gas) muscle by Succinic dehydrogenase (SDH) staining and ratio of oxidative fibers. (f) Representative images of gastrocnemius muscle by transmission electron microscopy and quantification of mitochondrial area. Data are presented as mean ± SEM and Student t tests were performed to compare control and Rosi‐treated group. (*p < 0.05, **p < 0.01)

Given that the aging process is accompanied by elevated inflammation in tissues such as fat and liver and by increased sarcopenia and muscle atrophy (Dalle et al., 2017; Kalyani et al., 2014), we performed gross pathology and histological analysis to assess the effects of chronic Rosi treatment started when mice reach 14 months of age. Immuno‐histochemical analysis using Sirius Red staining and an F4/80 antibody of WAT revealed, respectively, diminished fibrosis and decreased macrophage infiltration in the Rosi‐treated group (Figure 2a,b). Furthermore, treated mice showed decreased hepatic inflammation (Figure 2c) and diminished islet degeneration (Figure 2d).

Given the known role of Rosi in regulating mitochondrial function in muscle (Rong et al., 2007), we compared muscle fiber size and number and mitochondrial morphology of muscle obtained from control and treated mice. As shown in Figure 2e, SDH staining revealed increased oxidative fibers and electron microscopy analysis showed elevation in mitochondrial density in muscle of Rosi‐treated mice (Figure 2f). Overall, these results suggest that Rosi prevents age‐associated tissue inflammation, fibrosis, and atrophy in a number of metabolic tissues.

### 2.3 Rosiglitazone treatment relieves anxiety‐ and depression‐like symptoms and improves cognitive functionality

**FIGURE 3:**
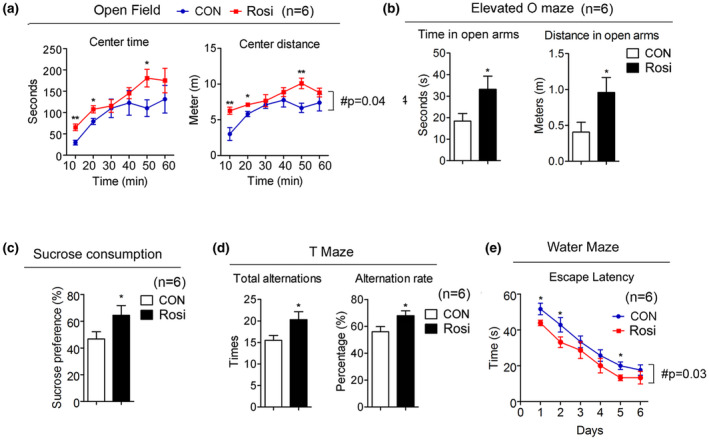
Rosiglitazone treatment is associated with decreased anxiety‐like behavior and depression‐like symptoms and improved cognitive function. (a–e) Analysis of 20‐month‐old mice after they were treated for 6 months either, with control or Rosi at 1 mg/kg/day (n = 6). (a) Open field analysis indicating time and distance. (b) Elevated O maze showing the length of time mice stay in the open arm and the distance. (c) Sucrose preference test. (d) Total alternation and alternation rate in the T‐maze test. (e) Water maze test. Data are presented as mean ± SEM. Student t tests were performed to compare control‐ and Rosi‐treated group (* *p* < 0.05, ** *p* < 0.01)

It has been previously reported that increased locomotor activity in mice may be a sign of anxiety‐ and depression‐like conditions (Seibenhener & Wooten, 2015). Given that Rosi‐treated mice showed reduced activity (Figure 1i), we assessed whether control and treated mice displayed differences in anxiety‐ and depression‐like behaviors. Behavioral studies revealed that 14‐month‐old mice receiving Rosi spent more time in the center area of the open field maze and in the open arm of the elevated zero maze compared to controls (Figure 3a,b), suggesting decreased anxiety‐like symptoms. Given the known association between stress and attenuated sucrose preference (Wang et al., 2018), we performed sucrose preference tests and demonstrated that Rosi‐treated mice consume more sucrose after exposure to stress than control mice (Figure 3c), suggesting a decrease in depression‐like symptoms in treated mice. The aging process is known to be accompanied by neurodegeneration and brain deterioration (Dillin et al., 2014); we therefore determined possible changes in learning abilities and memory in control and treated mice. Our data demonstrated that Rosi‐treated mice had improved cognitive function, as shown by their increased alternations in the T‐maze, and decreased escape latency in the water maze (Figure 3d,e).

### 2.4 Rosiglitazone reprograms the expression profile of genes involved in inflammation and mitochondrial function in aging metabolic tissues

**FIGURE 4:**
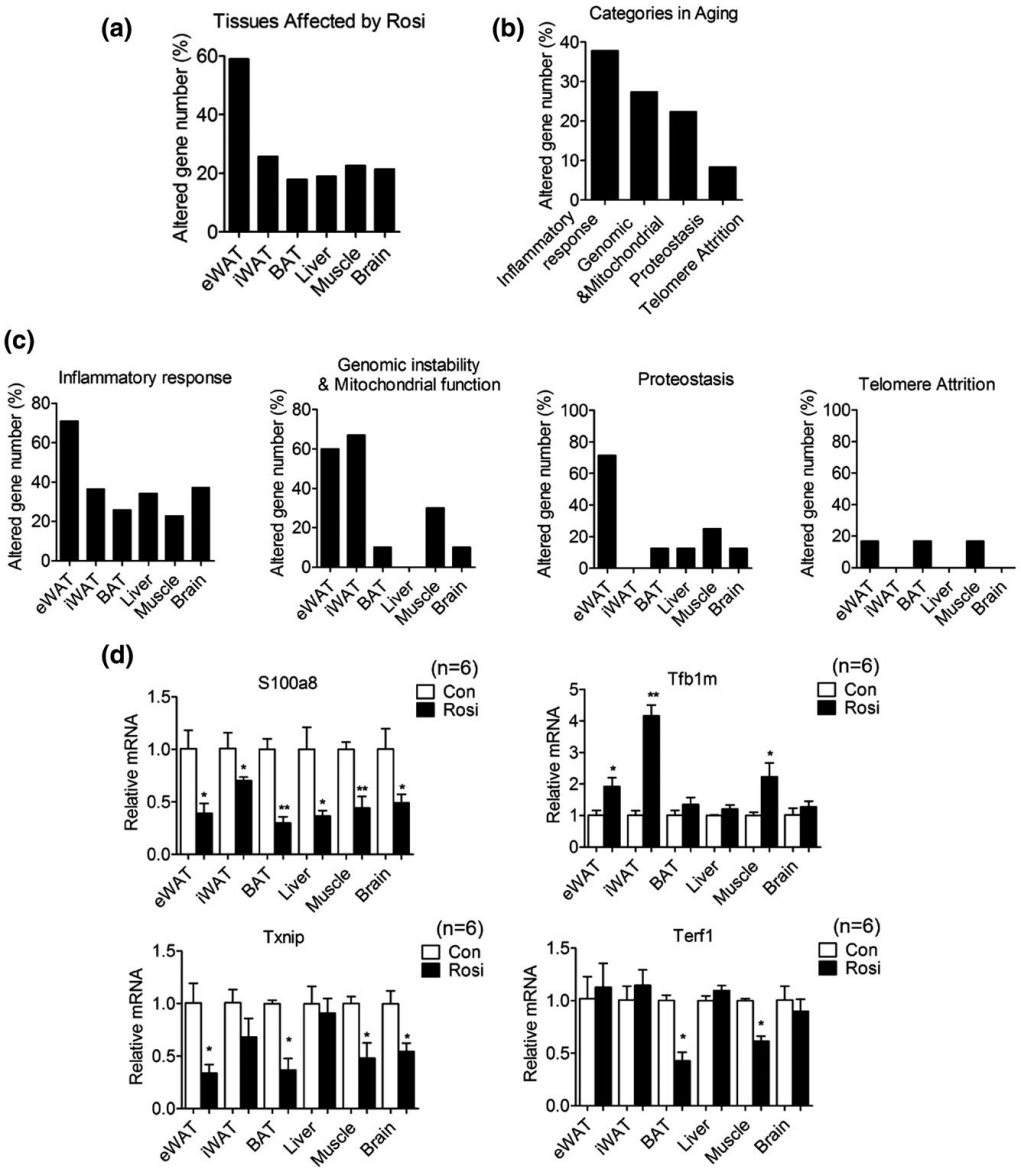
Chronic treatment with low doses of rosiglitazone alters the levels of expression of genes involved in inflammatory responses and in mitochondrial functionality. (a) Percentage of differentially expressed genes (DEGs) in adipose tissues, liver, muscle and brain in 20‐month‐old mice after they were treated for 6 months. (b, c) Distribution of modulated genes in different pathways and tissues. (d) Expression levels of representative genes of each category in multiple organs of 20‐month‐old mice after 6 months of treatment. Data are presented as mean ± SEM and Student t tests were performed to compare control and Rosi‐treated group (*p < 0.05, **p < 0.01, n = 6 per group)

To better understand the molecular mechanisms underlying Rosi's action in metabolic tissues, we performed unbiased analysis of gene expression in adipose depots, liver, muscle, and brain. These analyses revealed that mice undergoing Rosi treatment have the highest percentage of gene expression changes in eWAT compared to the other tissues analyzed (Figure 4a and Table S2). Analysis of average changes in gene expression demonstrated that factors involved in inflammatory responses, genomic stability, and mitochondrial functionality are the most differentially regulated genes in response to Rosi treatment in each of the tissues analyzed (Figure 4b). Specifically, Rosi‐treated mice showed decreased levels of inflammatory genes and increased expression of genes encoding for factors involved in mitochondrial functionality and genome stability. Furthermore, mitochondrial gene expression changes appeared to be WAT tissue‐selective, since minimal effects were observed in BAT, liver, and brain (Figure 4c). Validation of the differential expression of select genes confirmed the results obtained via the arrays (Figure 4d). Overall, our molecular analysis suggests that Rosi treatment is associated with decreased age‐associated dysfunction, possibly through Rosi effects in reducing inflammation in metabolic tissues and in preserving mitochondrial functionality.

### 2.5 Effects of long‐term low‐dose rosiglitazone treatment on cardiac function and skeletal system

**FIGURE 5:**
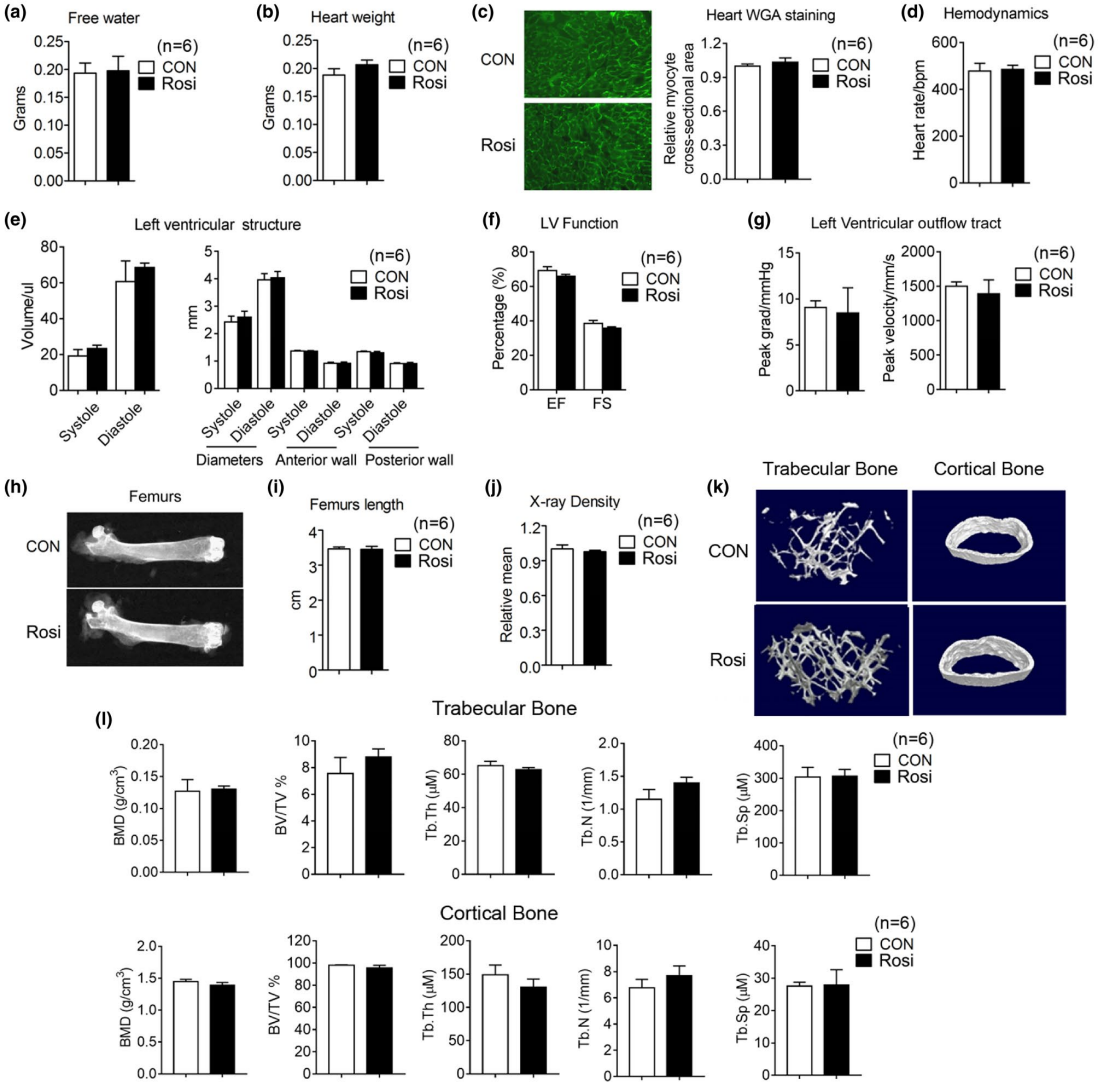
Limited adverse effects of long‐term rosiglitazone treatment at the low dose of 1 mg/kg/day on water retention, heart functionality, and bone density. (a–f) Analysis of 17‐month‐old mice after they were treated for 3 months with either control diet or Rosi‐containing diet (1 mg/kg/day) (n = 6). (a) Water content. (b, c) Heart weight and representative images of Wheat Germ Agglutinin (WGA) staining of heart. (d–g) Measurements of heart functionality include hemodynamics, left ventricular (LV) structure, LV function and LV outflow tract. (h) Representative images of femurs by X rays, (i) comparison of femur length, (j) measurements of X‐ray density, (k) micro‐CT reconstruction, and (l) parameters of trabecular and cortical bone. Data are presented as mean ± SEM

It has been previously reported that Rosi treatment of human subjects increases myocardial infarction, water retention, and bone fractures (Nissen & Wolski, 2007). To potentially reduce the incidence of issues arising from the long‐term use of Rosi, we exposed mice to lower doses than those previously used (Cellai et al., 2010). Comprehensive serum analysis revealed no significant differences except in glucose levels, which appeared to be significantly lower in Rosi‐treated mice (Table 1). Furthermore, as shown in Figure 5a, no significant differences in water retention between treated mice and controls were revealed by Echo MRI analysis. In vivo echocardiogram showed no cardiac abnormalities (Table 2), and measurements of heart weight and WGA staining of heart biopsies (Figure 5b–g) revealed no differences in heart volume and in left ventricular ejection fraction between groups, although a minor trend to an increase in these parameters was noticed. To assess adverse effects of Rosi also on the bone, we performed micro‐computed tomography and demonstrated that Rosi‐treated mice have comparable amounts of trabecular and cortical bone to controls (Figure 5h–l). These results suggest that chronic treatment of Rosi at low doses has limited adverse effects.

**TABLE 1:** Serum parameter analysis of control‐ and Rosi‐treated mice

Tests	CON	ROSI	*p*
Minerals			
Sodium (mmol/L)	157.0 ± 6.6	151.7 ± 4.6	0.13
Potassium (mmol/L)	9.95 ± 0.12	9.11 ± 1.42	0.19
Chloride (mmol/L)	116.2 ± 6.49	111.5 ± 7.79	0.29
Calcium (mmol/L)	2.31 ± 0.18	2.36 ± 0.14	0.59
Magnesium (mmol/L)	1.76 ± 0.31	1.56 ± 0.11	0.16
Phosphorus, inorganic (mg/dL)	9.48 ± 3.31	8.65 ± 1.64	0.59
Glucose (mg/dL)	152.2 ± 29.24	120.0 ± 14.17	0.03*
Lipids			
Total cholesterol (mg/dL)	87.0 ± 15.91	83.0 ± 24.23	0.67
Triglycerides (mg/dL)	37.0 ± 19.91	33.3 ± 10.84	0.71
Renal/proteins			
Urea nitrogen (BUN, mg/dL)	32.0 ± 9.96	30.0 ± 5.14	0.52
Creatinine (mg/dL)	0.35 ± 0.05	0.30 ± 0.31	0.95
Uric acid (mg/dL)	5.16 ± 2.56	4.30 ± 1.92	0.83
Albumin (g/dL)	3.08 ± 0.29	3.10 ± 0.61	0.74
Total protein (g/dL)	4.43 ± 0.49	4.52 ± 0.79	0.70
Enzymes			
Alkaline phosphatase (U/L)	120.2 ± 26.5	126.5 ± 49.3	0.78
Alanine aminotransferase (ALT, U/L)	38.67 ± 17.85	44.67 ± 16.31	0.55
Aspartate aminotransferase (AST, U/L)	115.50 ± 34.57	131.33 ± 48.91	0.41
Amylase (U/L)	3596.3 ± 1601.5	2879.0 ± 572.7	0.32
Creatine kinase (U/L)	1424.8 ± 471.9	1251.0 ± 307.9	0.46
Lactate dehydrogenase (U/L)	774.0 ± 276.07	695.7 ± 411.1	0.71

Note

Student t tests were performed to compare serum parameters of control‐ or Rosi‐treated mice of 20 months of age after 6 months of treatment. N = 6 per group. * symbol is used to highlight statistically significant values

**TABLE 2:** Heart parameters measured by Echo MRI

	CON	TZD	*p* value
Hemodynamics			
Heart rate, bpm	478.33 ± 33.13	485 ± 18.06	0.67
Left ventricular (LV) structure			
Volume; systole, ul	19.23 ± 8.74	23.36 ± 4.52	0.31
Volume; diastole, ul	60.65 ± 28.22	68.51 ± 5.96	0.52
Diameters; systole, mm	2.43 ± 0.21	2.60 ± 0.22	0.23
Diameters; diastole, mm	3.96 ± 0.23	4.04 ± 0.23	0.55
LV anterior wall; systole, mm	1.37 ± 0.02	1.36 ± 0.02	0.94
LV anterior wall; diastole, mm	0.92 ± 0.04	0.92 ± 0.05	0.73
LV posterior wall; systole, mm	1.34 ± 0.03	1.30 ± 0.05	0.27
LV posterior wall; diastole, mm	0.91 ± 0.03	0.91 ± 0.04	0.88
LV function			
Ejection fraction (EF, %)	69.25 ± 5.41	65.93 ± 2.49	0.30
Fractional shortening (FS, %)	38.53 ± 4.25	35.66 ± 2.13	0.28
Left Ventricular outflow tract			
Peak grad, mmHg	9.07 ± 1.77	8.48 ± 6.69	0.84
Peak velocity, mm/s	1499.83 ± 153.2	1390.4 ± 489.86	0.61

Note

Student t tests were performed to compare heart parameters in 17‐month‐old mice after they were treated with Rosi or control for 3 months, starting at 14 months of age. N = 6 per group.

### 2.6 Long‐term treatment with rosiglitazone extends lifespan in mice

**FIGURE 6:**
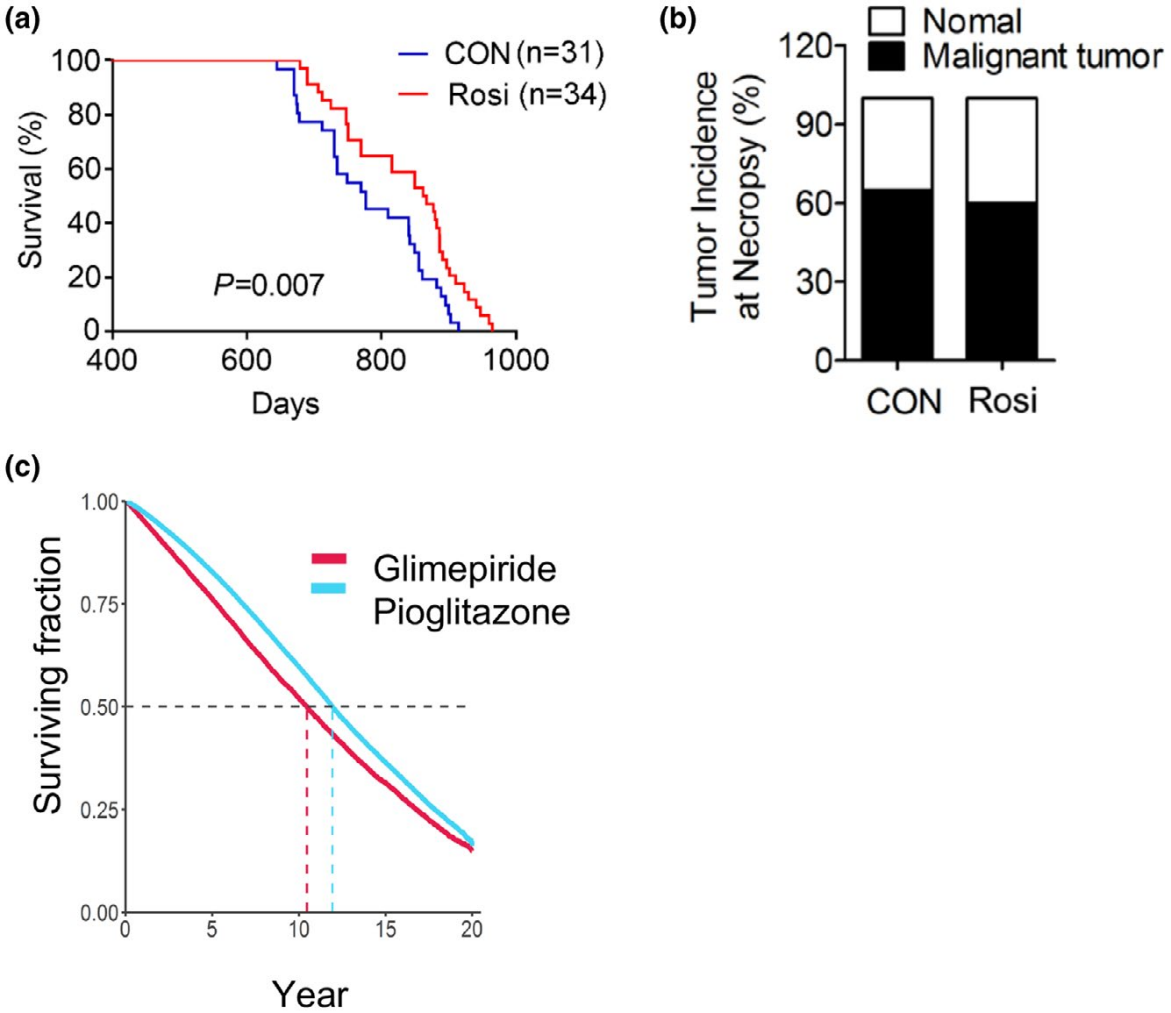
Chronic treatment with low doses of rosiglitazone (1 mg/kg) of 14 months of age male mice is associated with increased lifespan with no alterations in tumor incidence and survival on pioglitazone in comparison to glimepiride. (a) Longevity curve, (b) tumor incidence at necropsy in control and Rosi‐treated mice. Curves were plotted with the Kaplan–Meier method and compared using the log‐rank test (control group, n = 31 and Rosi‐treated group, n = 34). (c) Survival curve of patients of the Veterans Health Administration on pioglitazone or glimepiride

Given the effects of Rosi treatment on amelioration of the metabolic and cognitive functions we observed, we next determined whether exposure to Rosi may affect lifespan, even if the treatment is started in mice when they are 14 months of age and therefore have potentially already undergone some of the metabolic changes associated with aging. Our data demonstrate that chronic treatment with 1 mg/kg/day of Rosi started when the mice are 14 months old, extended median lifespan by 11% (Figure 6a). The median survival (864 days) was significantly increased in treated mice compared to the control group (777 days). Age is a major risk factor for the development of cancer; therefore, we evaluated the effects of Rosi treatment on the natural development of tumors. Rosi has been shown to have anti‐tumorigenic properties in lung, breast, and colon cancer through inhibition of cell growth and invasion and via induction of apoptosis in cancer cell lines (Fenner & Elstner, 2005). Our analysis of 50 pathology reports of mice in the longevity cohort determined by necropsy at the time of death revealed a trend in delayed development of spontaneous tumors, in Rosi‐treated mice (Figure 6b and Figure S5). These data suggest that Rosi treatment is associated with increased lifespan and that the anti‐cancer properties of Rosi may not contribute to the effects of Rosi on longevity and healthspan.

### 2.7 Pioglitazone correlates with increased lifespan in human subjects

In order to assess whether the longevity effects observed in mice in response to Rosi treatment may correlate with outcomes in humans treated with PPARγ agonists, we performed a retrospective survival analysis and reviewed electronic medical record data to assess the effects of the PPARγ agonist Pio on longevity (Table S3). We chose to assess the effects of Pio, given that it has been the thiazolidinedione of choice continually for the last 20 years, instead of Rosi, which in the USA was temporarily subject to prescribing restrictions due to safety concerns. As shown in Figure 6c, the Kaplan–Meier curve comparing the effects on lifespan of Pio to the stimulator of insulin secretion glimepiride, which is extensively prescribed for the treatment of insulin resistance, demonstrated that patients on Pio have increased survival compared to those on glimepiride. These data suggest that PPARγ ligands may have life‐extending effects both in preclinical studies and in human subjects.

## 3 DISCUSSION

Aging is associated with metabolic dysfunction and increased chronic, low‐grade inflammation. In addition, aging leads to tissue damage and the onset of a constellation of chronic diseases in multiple organs. Recently, insulin‐sensitizing and anti‐inflammatory drugs have been evaluated for their anti‐aging effects, given that it has been shown that they may increase physical and metabolic performance, cognitive function, muscle endurance, insulin sensitivity, and lifespan, in worms, mice, and rats, mimicking some of the benefits of calorie restriction (Li et al., 2014; Wu et al., 2016). Given the known insulin‐sensitizing and anti‐inflammatory properties of PPARγ ligands, here we investigated the effects of TZDs in aging. Our data demonstrate that Rosi leads to physiological, morphological, and molecular changes in a number of metabolic organs, particularly in fat and muscle. Since these two tissues undergo significant atrophy in aging (Tyrovolas et al., 2016), our results suggest that Rosi maintains fat tissue homeostasis by improving glucose uptake, decreasing fibrosis and immune cell infiltration, and increasing muscle integrity, as shown by the compact appearance of muscle fibers visualized by electron microscopy in Rosi‐treated aging mice. The role of Rosi in preventing tissue atrophy is consistent with published reports demonstrating TZDs effects on alleviating adipose and muscle wasting in cancer cachexia (Tyrovolas et al., 2016) and in promoting insulin sensitivity in muscle of aged rats (Song et al., 2010).

Rosi treatment appears to decrease inflammation also in liver and to maintain the structure of pancreatic islets, whose architecture is normally disrupted during aging (Liu et al., 2013). It remains to be determined whether the changes in organ functionality of Rosi‐treated mice are due to the direct effects of Rosi or are indirect, due to overall improved metabolic functionality, and if they are PPARγ‐dependent or PPARγ‐independent. It is plausible that PPARγ may be the mediator of Rosi's action in a number of tissues, given that mice with specific loss of PPARγ in fat, muscle, liver, pancreas, brain, or macrophages show metabolic dysfunction and fail to respond to Rosi treatment (Ahmadian et al., 2013).

It has been previously reported that Rosi increases the risk of cardiovascular disease and bone fractures (Kahn et al., 2006; Nissen & Wolski, 2007) and that, because of these side effects, Rosi was subjected to prescribing restrictions in the USA during 2011–2013. Taken this into consideration, we treated healthy 14‐month‐old mice with doses of Rosi lower than those previously used in preclinical studies and demonstrated that osteopenia and cardiomyopathy are absent after 6 months of treatment. These data suggest that chronic treatment with Rosi at low doses improves metabolic parameters and cognitive functionality, without any overt adverse effects.

Our molecular analysis revealed that eWAT underwent the highest degree of changes in response to Rosi treatment, specifically in the expression levels of genes encoding for factors governing inflammation. Given that accumulation of macrophages and of other immune cells in eWAT has been linked to age‐associated metabolic dysfunction (Lumeng et al., 2011), it is conceivable that Rosi could exert its anti‐aging effects through PPARγ‐mediated suppression of age‐related inflammation. Our analysis found that the expression of genes known to be associated with aging phenotypes, such as S100a8 and Txnip, is significantly down‐regulated in Rosi‐treated tissues, consistent with previous data demonstrating that these genes are modulated by TZDs in animal models of insulin resistance and diabetic nephropathy (Qi et al., 2009).

Our results suggest that chronic treatment with low doses of Rosi may represent a possible strategy to prevent, or slow down, the natural progression of age‐driven brain deterioration. It can be envisioned that Rosi‐mediated improvement in glycemic control and increased cognitive functionality may stem, at least in part, from the effects of Rosi on its cellular target, the nuclear receptor PPARγ, given that PPARγ plays a key role in glucose metabolism, and because it is expressed in neurons, astrocytes, and oligodendrocytes (Villapol, 2018) and since its loss of function increases the susceptibility to brain damage (Zhao et al., 2009). Furthermore, decreased expression of PPARγ in neuronal cells of young mice has been associated with spatial cognitive deficits, anxiety‐ and depression‐like behaviors, and neuro‐inflammatory effects, which can be reversed by Rosi (Paintlia et al., 2006; Zhou et al., 2016). Rosi penetrates the blood–brain barrier; therefore, it is conceivable that it may exert also its anti‐inflammatory and neuroprotective effects on microglia via PPARγ inhibition of pro‐inflammatory cytokines through NF‐kB (Carta & Pisanu, 2013). This possibility is supported by our studies indicating that Rosi decreases the expression of genes encoding for factors involved in inflammation in the brain.

Our data also demonstrate that low doses of Rosi improve cognitive function in mice with no genetic predisposition to Alzheimer disease (AD). Published studies have previously shown beneficial effects of higher doses of Rosi (5–30 mg/kg/day) in animal models of AD (Carta & Pisanu, 2013), but no reports to date have assessed the effects of low doses of TZD in protecting from the natural development of AD. Our results also highlight the beneficial effects of Rosi on depression‐ and anxiety‐related behaviors in aging. Although to date few studies have addressed the role of PPARγ agonists as antidepressants (Domi et al., 2016), our data suggest their potential use to treat mood disorders. Given that the PPARγ coactivator PGC1α protects from stress‐induced changes associated with depression through the regulation of kynurenine aminotransferase (Agudelo et al., 2014), it can be envisioned that PPARγ and PGC1α may cooperate in regulating the levels of kynurenine, given the presence of PPREs in the kynurenine aminotransferase gene promoter (Agudelo et al., 2014).

Our data demonstrate that chronic treatment with low doses of Rosi has beneficial effects on longevity. While the mean lifespan of 777 days observed in the control mice in our cohort is in line with the mean survival age previously shown to occur at NIH animal facilities (Moskovitz et al., 2001), it appears to be lower than the average mean lifespan recorded for C57BL/6J on the JAX Mouse Phenome Database. This difference may be attributed to differences in husbandry conditions and may be dependent on a number of factors and variables such as ambient temperature, chow formulation, degree of exposure to pathogens, and microbiota. Further detailed studies comparing the effects of Rosi versus those of control diet on C57BL/6J mice performed in other animal facilities will provide additional information on the magnitude of the advantage in average survival conferred by Rosi and will permit the dissection of facility‐dependent variables that may overall influence lifespan. Our study was performed in C57BL/6J male mice and did not include the evaluation of the effects of Rosi on females nor on other strains. Given that it has been shown that both sex and genetic background are important contributors of longevity outcomes, future studies will be needed to evaluate the effects of Rosi on longevity and healthspan also in females and in mixed mouse strains.

To our knowledge, no study has evaluated the long‐term impact of Rosi on lifespan in mice without any predisposing genetic alterations. Our retrospective studies comparing longevity in subjects receiving an antidiabetic compound of a different category and with distinct mode of action suggest that PPARγ ligands could increase survival also in humans. Given that Pio in the USA has never been subjected to prescribing restrictions over safety concerns, we have been able to perform the evaluation of the consequences of this TZD on survival over 20 years. Whether our retrospective studies can also infer the effects of TZD on healthspan and overall longevity in healthy subjects remains to be determined through a prospective study. It is worth noting that previous reports on elderly AD patients treated with TZDs showed increased delayed recall and selective attention, and decreased plasma beta‐amyloid levels (Zhou et al., 2014).

In conclusion, in this study we demonstrated that chronic treatment with low doses of Rosi counteracts the metabolic derangement and cognitive dysfunction occurring in aging mice without side effects and that the long‐term use of TZDs correlates with longer lifespan in mice and increased survival in humans. Overall, our data support the use of low‐dose TZDs as possible novel pharmacological interventions to counteract aging, promote healthspan, and increase longevity.

## 4 METHODS

### 4.1 Mice

C57BL/6J mice were purchased from JAX laboratory and maintained in a humidity and temperature‐controlled (22–24°C) animal facility with a 12 hr light/dark cycle following the guidelines of the National Institute of Diabetes, Digestive and Kidney Diseases (NIDDK) and of the New York University Animal Care and Use Committee. The circulating air in the housing facility was HEPA filtered and the drinking water was not acidified. Experimental male mice were all housed in the same room of the animal facility and serologic tests were conducted once a month on sentinel mice to prove lack of viral infection. No infections occurred in the animal colony during the experimental period reported. Only male mice at 14 months of age or older were used in this study. For treatment, chow diet (NIH 07) was mixed with, or without, Rosi and given at 1 mg/kg of mouse weight/day. Fat mass, lean mass, and water retention were measured by NMR (Echo medical system). Metabolic parameters, such as oxygen consumption, food intake, and locomotor activity, were measured via the CLAMS system (Columbus instruments system) in 20‐month‐old mice, after 14‐month‐old mice were exposed for 6 months to control or Rosi‐containing diet. Insulin sensitivity was measured by GTT and ITT in 17‐month‐old male mice, after they were treated for 3 months with either control or Rosi diet, as previously described (Ayala et al., 2010). Briefly, for GTT analysis, mice were fasted for either 6 hours or O/N and injected intraperitoneally with glucose in saline solution (2 g/kg) and plasma glucose levels were measured from tail blood at 0, 15, 30, 60, 90, and 120 min after glucose injections (AlphaTrak Blood Glucose Monitor System, Abbott). For ITT analysis, mice received an intraperitoneal injection of insulin in saline solution (1 mU/kg) and plasma glucose levels were also measured at 0, 15, 30, 60, 90, and 120 min. For sucrose testing, regular water bottles were removed from each mouse cage and replaced with two graduated plastic bottles containing pre‐measured 1% sucrose solution and pre‐measured water, separately. The amount of sucrose solution and water consumed was measured in time periods ranging from 4 to 24 hr. The percent preference for the sucrose solution provided an indirect measure of depression‐like symptoms. For open field testing 20‐week‐old male mice were gently placed in a 16 × 16 × 16 Perspex arena viewing chamber and videotaped for periods ranging from up to 60 min. The mouse behavior was analyzed by the patterns of movement into the center of the chamber and for the length of time the mice remained around the edges of the arena. For Elevated Zero Maze, we used elevated circular runways either enclosed by walls or unenclosed, with an open area with 66 cm in width and 84 cm in height. Each mouse was placed on the runway and allowed to explore the maze for 5 minutes. The ratio of time the mouse spent in the open areas of the maze versus that spent in the enclosed parts was used as a measure of anxiety‐like symptoms. For Spontaneous T‐maze studies, each mouse was placed in a three‐arm maze for five minutes and allowed to spontaneously explore and travel freely in the apparatus. The number of arm's visits performed by each mouse during five minutes was recorded, and the alternation rate was analyzed. These values were used for the assessment of cognitive function.

### 4.2 Lifespan and cancer incidence analysis

Lifespan monitoring of male mice was performed as previously described (Ma et al., 2014). Briefly, 14‐month‐old male mice used to study longevity and cancer incidence were kept up to five per cage and were not used for any other metabolic or physiological analyses. The date of their death was recorded as the day that either the mice were found dead in the cage or when euthanasia was performed based on the veterinary technician recommendations due to severe discomfort. Survival curves were plotted with Kaplan–Meier methods, and statistical analysis was performed with log‐rank test by GraphPad. Pathological analysis of malignant tumors was performed by a veterinary pathologist of the Division of Veterinary Resources of the NIH on dissected tissues (see Section 4.4 below) and Fisher's exact test was used to compare control or Rosi‐treated mice.

### 4.3 Primary cells preparation and measurement of D‐[^14^C]‐glucose uptake

Mice were anesthetized via an intraperitoneal injection of pentobarbital sodium (65 mg/kg body weight), and muscle (gastrocnemius) and adipose tissues (inguinal and visceral) were excised. Skeletal muscle cells and adipocytes were isolated as previously described with some deifications. Briefly, excised tissues were washed twice in Hank's Buffered Salt Solution (HBSS) (Invitrogen) and then minced into a coarse slurry. The minced tissues were then digested with either a Ca‐free 0.25% trypsin solution containing EDTA (Gibco) for 20 min (for skeletal muscle cells) or with a Krebs‐Ringer bicarbonate HEPES (KRBH) buffer with 0.1% collagenase‐type I (Gibco) for 40 min (for adipocytes) in a 37°C water bath with gentle agitation. The digestion solution was filtered through a 100 μm nylon mesh (Falcon) and centrifuged for 5 min at 800 g. Cell pellets were then resuspended in Dulbecco's modified Eagle's medium (DMEM) (Invitrogen) and incubated for 30 min with gentle agitation. Glucose uptake assay was performed according to previously described protocols. Briefly, cells were washed twice in KRBH buffer containing 0.1% BSA and then resuspended in the wash buffer at 20% cytocrit. The cell suspension was equilibrated for 10 min at 37°C with gentle agitation and then stimulated with insulin (100 nM) for 15 min. Next, the ^14^C‐labeled glucose medium consisting of 5 mM D‐glucose (containing 2.5 μCi/ml D‐[14C] glucose (Perkin Elmer) was added and incubated for 5 min at 37°C. The uptake was terminated by adding cytochalasin B (Solarbio) and washed three times with KRBH buffer containing 0.1% BSA. Finally, the cell‐associated radioactivity was measured using the scintillation cocktail medium Ultima Gold XR (Perkin Elmer) in a Tri‐Carb 4910TR Liquid Scintillation Analyzer (Perkin Elmer). The non‐specific signal was determined in control samples containing cytochalasin B without^14^C‐labeled glucose medium. Glucose uptake was normalized for DNA content.

### 4.4 Histological analysis

Tissues dissected from control‐ or Rosi‐treated mice were fixed in 10% neutral‐buffered formalin (Sigma) and embedded in paraffin. Tissue sections of 5 μm thickness were stained with hematoxylin and eosin (Histoserv). Frozen sections of gastrocnemius muscle were stained with succinic dehydrogenase (SDH) for oxidative fiber assessment (Histoserv) and wheat germ agglutinin (WGA) (Histoserv) for fiber quantification, respectively. Slides were analyzed using a microscope (Olympus) at the indicated magnification and images were captured by a digital camera (Olympus). The sizes of adipocytes were determined with ImageJ software. Specifically, for quantification, five random fields of H&E stained eWAT and iWAT of 6 mice per group were quantified and analyzed. Necropsies were performed by the NIH DVR Department at the NIH by removing individual organs, following the pathology procedure described above.

### 4.5 Transmission Electron Microscopy (TEM) analysis

For TEM analysis, dissected gastrocnemius muscle was cut into pieces of 1 mm size and placed in fixing solution containing 0.1 M phosphate buffer, 2% paraformaldehyde, and 2.5% glutaraldehyde (EM facility, National Cancer Institute, NCI). Fixed muscle tissue was subsequently washed and stored in 0.1 M cacodylate buffer. TEM was imaged in a Hiachi 7650 TEM operating at 80 kV. Images were taken with a digital camera from Advanced Microscopy Techniques and quantification analysis was performed with Image J (version 1.44; NIH).

### 4.6 3‐Dimensional Micro‐computed Tomography (CT) analysis

For Micro‐CT, the femur bone taken from male mice either control‐ or Rosi‐treated for 3 months was fixed in 10% neutral‐buffered formalin (Sigma) and scanned at a resolution of 9 µm pixels. The region analyzed was below the growth plate, from 720 μm (80 image slices) to 1620 μm (180 image slices). Trabecular and cortical bone parameters of the femur were calculated using CTan software (Bruker micro‐CT), and 3D reconstruction images were generated with software CTvol (Bruker micro‐CT).

### 4.7 Gene expression analysis

Total RNA was extracted with TRIzol (Invitrogen), according to procedures described previously (Verma et al., 2020), from iWAT, eWAT, and BAT, liver, brain, and muscle tissues of 17‐month‐old mice, after three months of treatment with either a control diet or a diet containing Rosi. RNA was reverse transcribed into cDNA (First Strand cDNA Synthesis Kit, Roche), according to the manufacturers’ instructions. For unbiased gene expression analysis of tissues obtained from control‐ and Rosi‐treated mice, we used Mouse RT^2^ Profiler™ PCR Array for Aging (Qiagen, PAMM‐178Z) for ABI PRISM 7900HT sequence detection system (ABI), using SYBR green (Roche). For validation of array studies and targeted analysis, we used the following primers: S100a8, Forward: AAATCACCATGCCCTCTACAAG; Reverse: CCCACTTTTATCACCATCGCAA; Tfb1m, Forward: CGGGAGATC ATTAAGTTGTTCGG; Reverse: GCCCAGGA CCCACTTCATAAA; Txnip, Forward: TCTTTTGAGGTGGTCTTCAACG; Reverse: GCTTTGACTC GGG TAACTTCACA; Terf1, Forward: CAGCAGTCTACAGA AACTGAACC; Reverse: ACTGAAATCTGATGGAGCACG; UCP1, Forward: GGCCCTT GTAAACAACAAAATAC; Reverse: GGCAACAAGAGCTGACAG TAAAT; Dio2, Forward AATTATGCCTCGGAGAAGACCG; Reverse: GGCAG TTGCC TAGTGAAAGGT.

### 4.8 Triglycerides analysis

100 mg of tissue was homogenized in phosphate‐buffered saline and lipids were extracted using a chloroform:methanol mix (2:1) and 0.1% sulfuric acid. The aliquot of the organic phase was collected, dried, and resuspended. Triglycerides content was then determined using commercially available kits (Sigma), following the manufacturer's instructions and data normalized to the respective tissue weight.

### 4.9 Retrospective survival analysis

We performed a retrospective survival analysis based on de‐identified electronic medical record data from the United States Veterans Health Administration (VHA) corporate data warehouse made available to us as part of the VA Obesity Medication Use Study (MIRB #1733), approved by the Research & Development committee and the Institutional Review Board at the Manhattan campus of the VHA New York Harbor Healthcare System. We focused on a comparison between subjects receiving Pio and the Sulfonylurea glimepiride, using an active comparator/new user approach. Of the TZD drugs used to treat insulin resistance, we chose to analyze the effects of Pio, given that this compound is the only PPARγ agonist that has been used without special restrictions over the course of the last 20 years in the United States. We selected glimepiride as a comparator because is an insulin secretagogue with a distinct mode of action from PPARγ agonists and because, similarly to pioglitazone, it is used almost exclusively for the treatment of type 2 diabetes. We queried the database to identify prescriptions for the medications of interest documented in the pharmacy records as released to the patients and not returned to stock. For each patient, we estimated the treatment start date to be the earliest day of release of the medication by the pharmacy. We queried the same database for dates of death. When not documented, we right‐censored survival status on the last date of vital sign documentation, therefore considering patients as lost to follow‐up after that date. In total, we identified 190590 subjects who were prescribed Pio and 47987 who were prescribed glimepiride (Table S3). We used non‐parametric approaches to estimate and compare survival after medication start date, without attempting to adjust for other factors, by means of Kaplan–Meier statistics and log‐rank testing, using R, a language and environment for statistical computing, version 3.6.1.

### 4.10 Statistical analysis

Student's t test was used for comparison between two mice groups and performed using GraphPad Prism software. The repeated measurements of ANOVA were performed with SPSS software to analyze the changes of different parameters over time. *p* < 0.05 was considered as statistically significant. Results are shown as mean ± SEM.

## CONFLICT OF INTEREST

The authors declare no conflict of interest.

## AUTHOR CONTRIBUTIONS

X.M., L.X., N.V., L.P., S.S., A.M.J., designed and performed experiments and analyzed results. J.A., P.L. designed experiments. J.Q., M.G., X.P., M.A., A.N., D.S., F.S., C.L., L.L., S.Z., J.L., and Z.D. performed experiments and analyzed results. J.P. analyzed human databases. X.M., L.X., and E.M. wrote the manuscript. E.M. conceived the project and coordinated its execution. All authors commented, edited and approved the manuscript.

## Supporting information

Fig S1

Fig S2

Fig S3

Fig S4

Fig S5

Table S1‐S3

## Data Availability

The data that support the findings of this study are available from the corresponding author upon reasonable request, however access to patient data is restricted.
