# Supplementary figures and images for: PPARγ agonists delay age‐associated metabolic disease and extend longevity
Source: Aging Cell. 2020 Nov 21;19(11):e13267. doi: 10.1111/acel.13267 (PMC7681041; doi:10.1111/acel.13267)

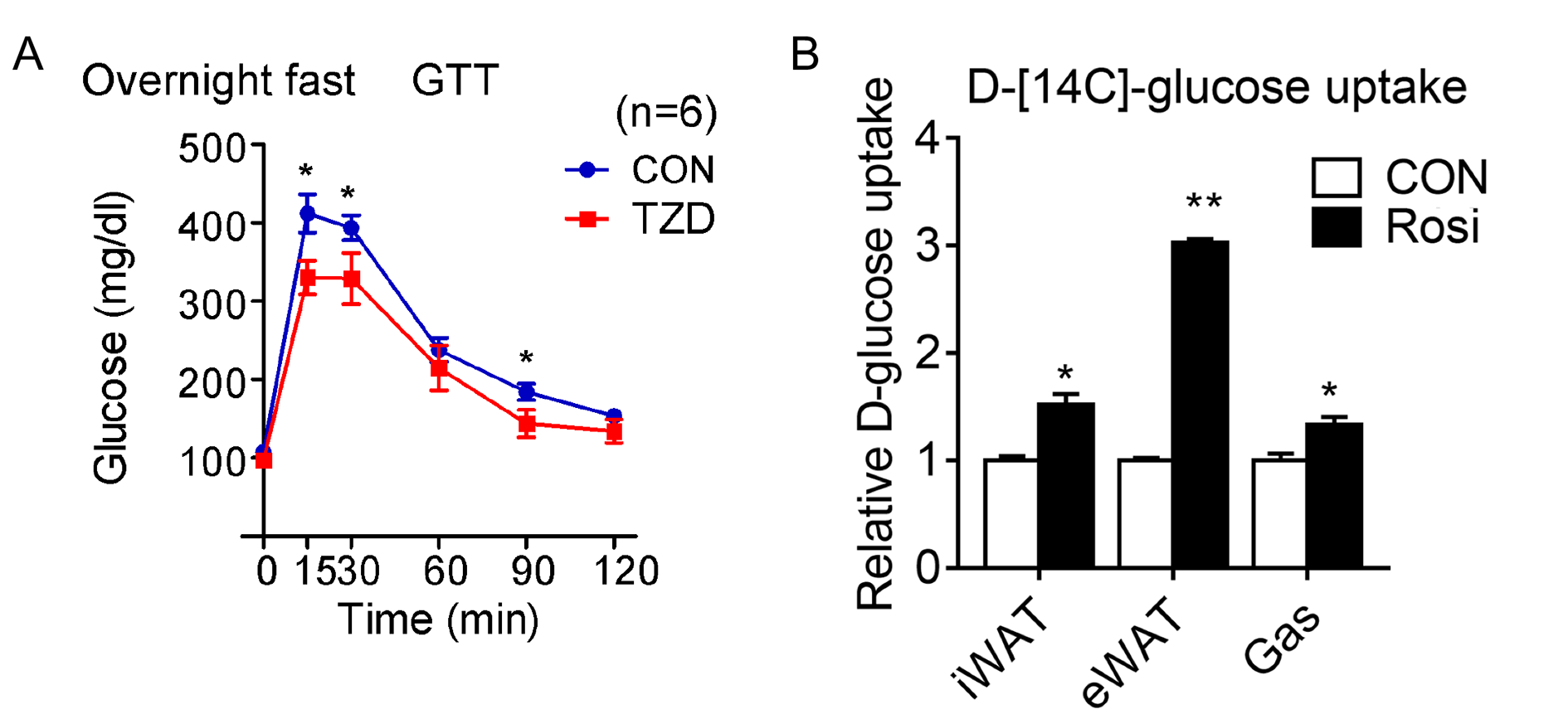

Supplement: Supplementary file 1 — Fig S1 [file ACEL-19-e13267-s001.tif]

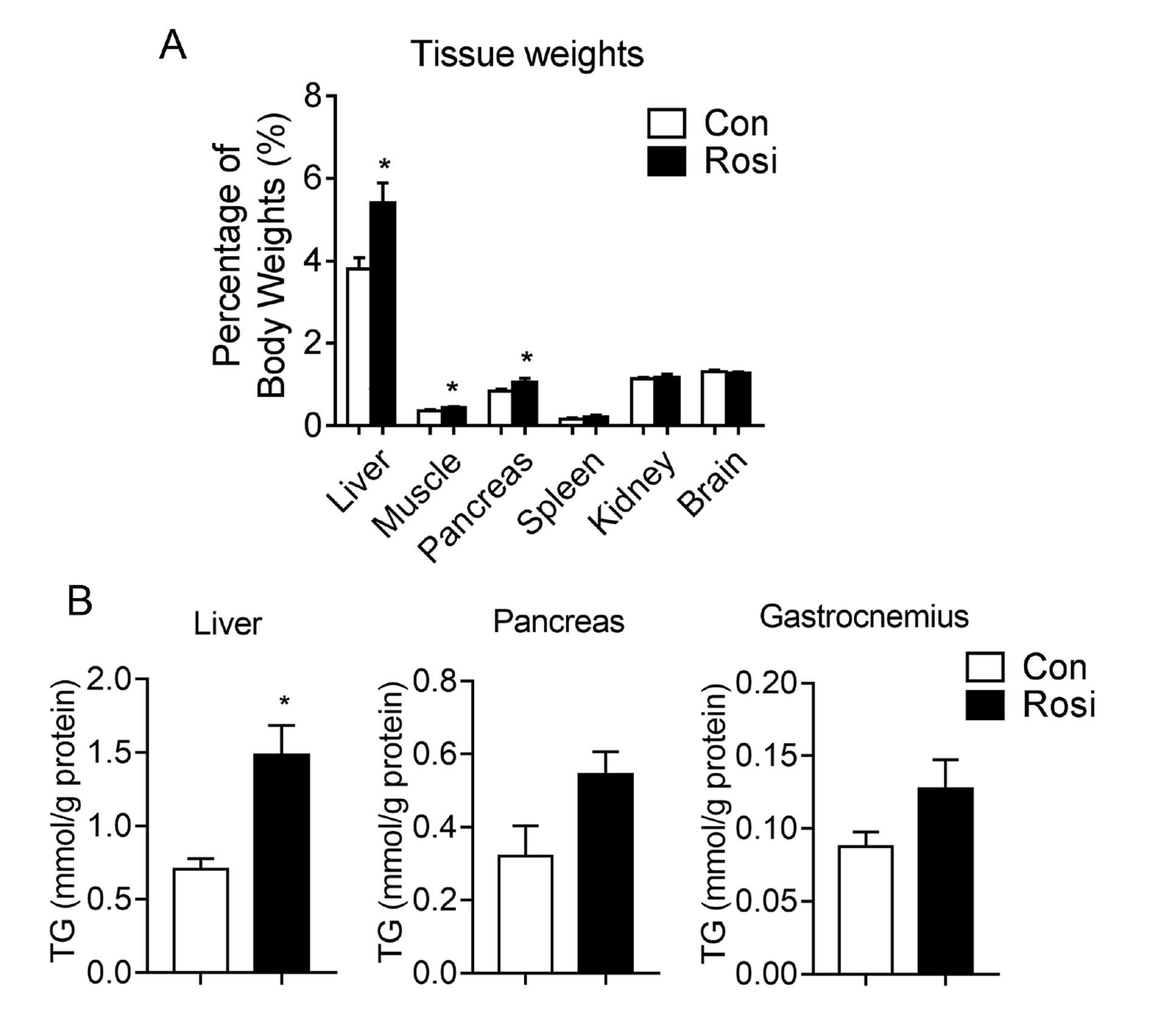

Supplement: Supplementary file 2 — Fig S2 [file ACEL-19-e13267-s002.tif]

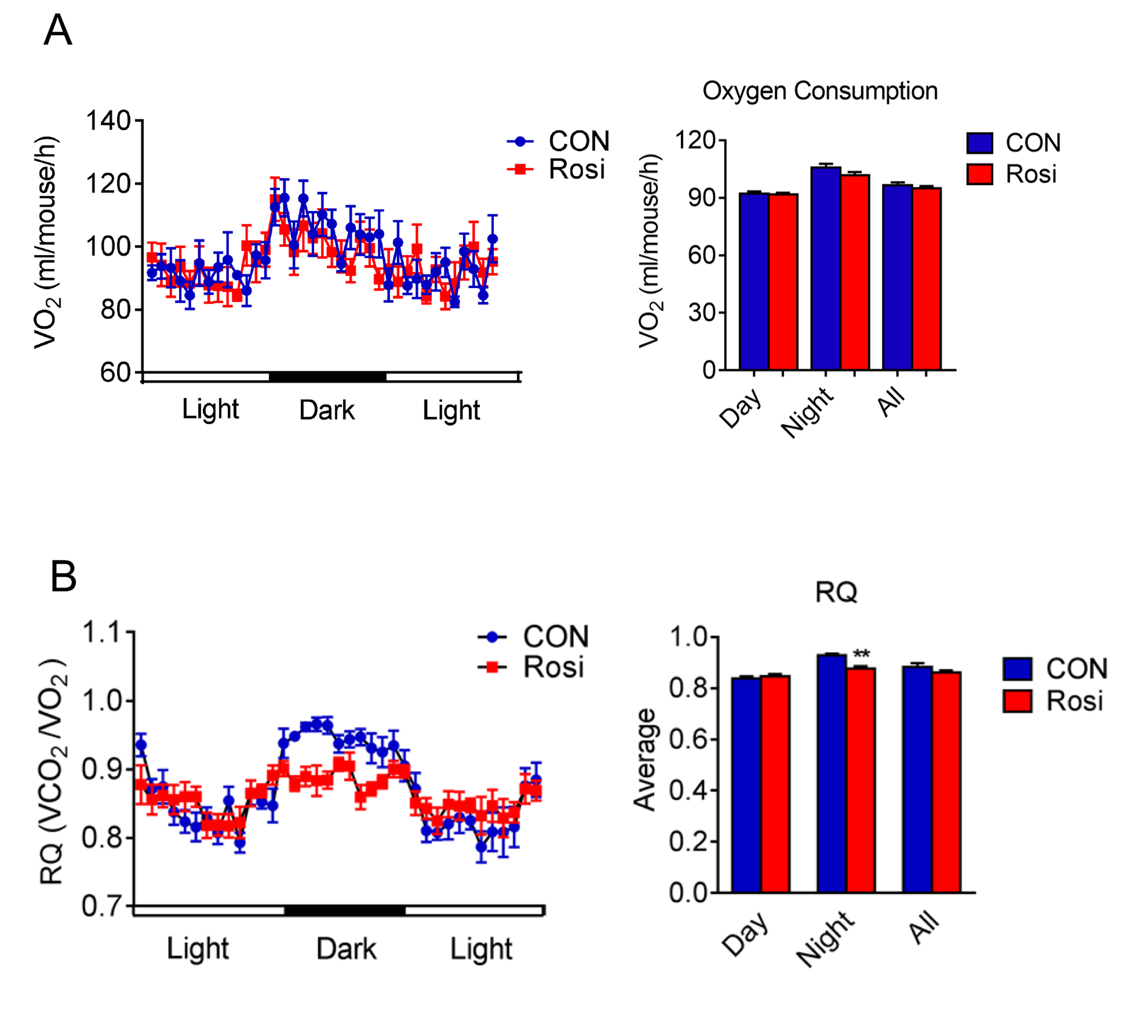

Supplement: Supplementary file 3 — Fig S3 [file ACEL-19-e13267-s003.tif]

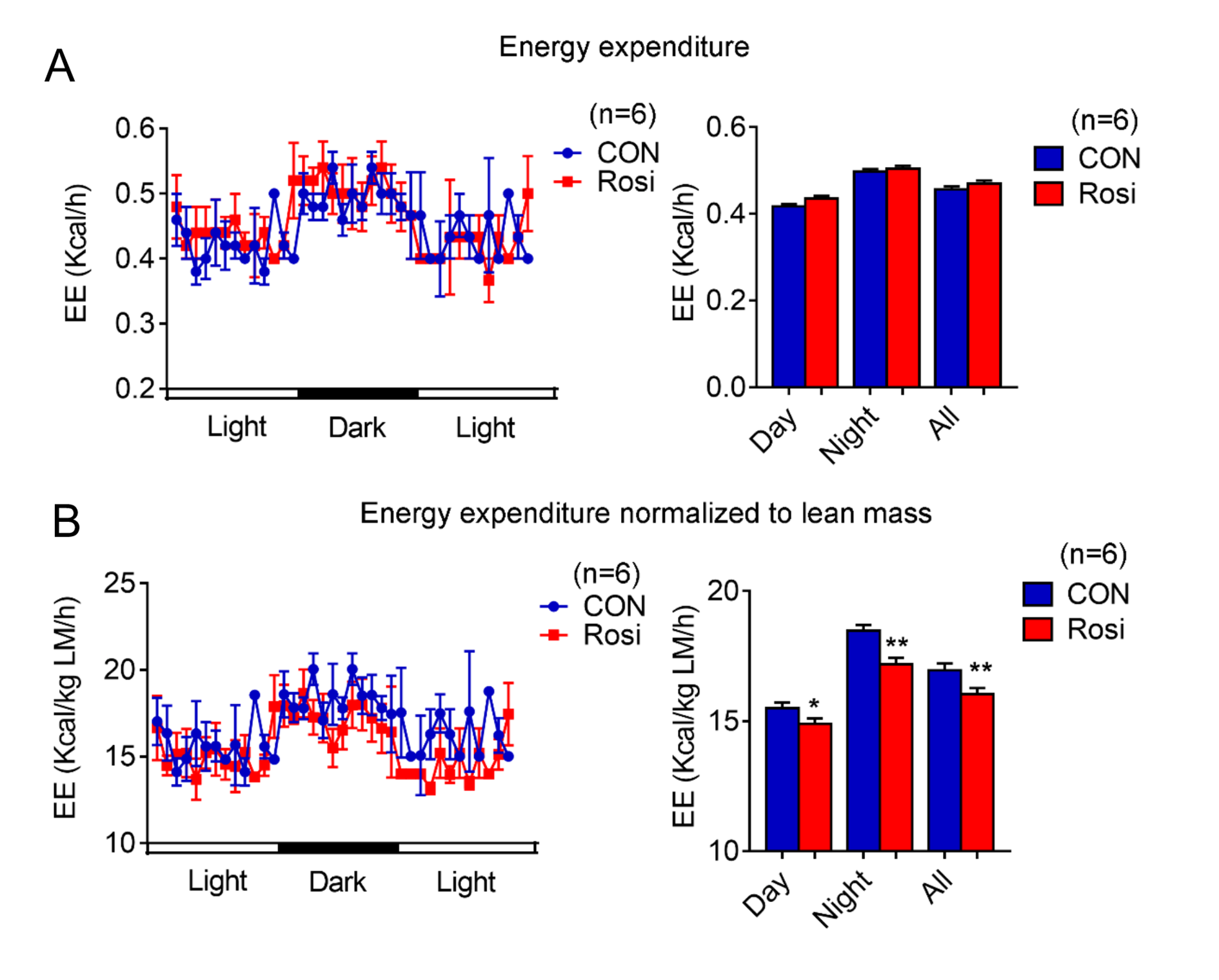

Supplement: Supplementary file 4 — Fig S4 [file ACEL-19-e13267-s004.tif]

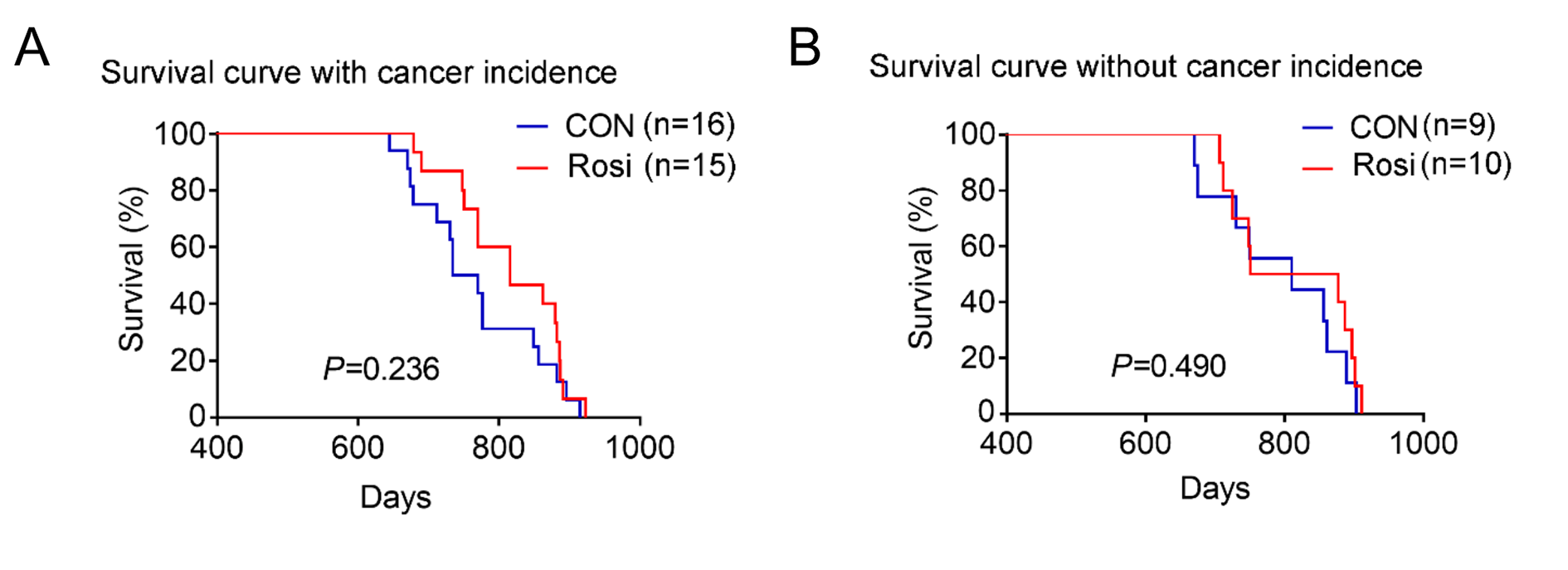

Supplement: Supplementary file 5 — Fig S5 [file ACEL-19-e13267-s005.tif]
