# Supplementary material for: PPARγ agonists delay age‐associated metabolic disease and extend longevity
Source: Aging Cell. 2020 Nov 21;19(11):e13267. doi: 10.1111/acel.13267 (PMC7681041; doi:10.1111/acel.13267)
Supplement: Supplementary file 6 — Table S1‐S3 [file ACEL-19-e13267-s006.docx]

**TABLE S1. Types and rates of neoplasia in control- or Rosi-treated aging mice.**

| **Neoplasia** | **Control** | **Rosi** |
| --- | --- | --- |
| Hepatocellular carcinoma | 4 | 2 |
| Lung adenocarcinoma | 3 | 1 |
| Histiocytic sarcoma | 5 | 7 |
| Hematopoietic neoplasia | 4 | 6 |
| Harderian gland adenoma | 0 | 1 |
| Oral squamous | 0 | 1 |
| Total neoplasia | 16 | 15 |
| Total autopsies | 25 | 25 |
| Cancer Incidence | 64% | 60% |
| Metastatic carcinoma | 3 | 2 |

**TABLE S2. Aging PCR-array results**

| Gene | eWAT | iWAT | BAT | Liver | Muscle | Brain |
| --- | --- | --- | --- | --- | --- | --- |
| Angel2 | - | - | 0.79 | 1.26 | 1.08 | 1.06 |
| Anxa3 | 0.51** | 0.97 | 0.91 | 0.81 | 0.94 | 0.92 |
| Anxa5 | 0.63* | 1.28 | 0.91 | 0.53** | 0.78 | 1.11 |
| Arid1a | - | - | 0.81 | 0.96 | 0.94 | 1.01 |
| Arl6ip6 | 1.03 | 1.36 | 0.96 | 1.14 | 0.75 | 0.98 |
| Bub1b | - | - | 0.76 | 0.77 | 0.85 | 0.94 |
| C1qa | 0.67* | 0.28** | 1.03 | 1.03 | 0.91 | 0.71* |
| C1qb | - | - | 1.05 | 1.15 | 1.04 | 0.92 |
| C1qc | 0.38** | - | 1.20 | 1.19 | 0.79 | 0.86 |
| C1s1 | - | 0.24** | 0.97 | 1.09 | 1.05 | 0.38* |
| C3 | 0.49* | 1.15 | 0.82 | 0.59* | 0.95 | 0.84 |
| C3ar1 | 0.85 | 0.19** | 0.80 | 1.12 | 0.95 | 0.77* |
| C4a | 1.15 | 0.46** | 0.66* | 0.70* | 0.60* | 0.62* |
| C4b | - | - | 0.64* | 0.72* | 1.06 | 0.82 |
| C5ar1 | - | - | 1.02 | 0.92 | 1.04 | 0.88 |
| Calb1 | 0.75 | 0.98 | 0.73 | 0.17** | 0.81 | 0.68* |
| Casp1 | 0.49** | 0.73 | 0.74 | 0.83 | 0.42** | 0.92 |
| Ccr1 | 0.38** | - | 0.71 | 0.39** | 0.81 | 0.55* |
| Cd14 | - | - | 1.02 | 0.51* | 1.17 | 0.47* |
| Cd163 | 0.24** | - | 1.12 | 0.32** | 0.98 | 0.82 |

| Cdkn1c | 0.55* | 1.40 | 0.73 | 0.42** | 1.06 | 1.27 |
| --- | --- | --- | --- | --- | --- | --- |
| Cfh | - | - | 1.47* | 1.08 | 1.49* | 0.86 |
| Cfhr1 | 0.63** | 0.78 | 0.65* | 0.71* | 0.66** | 0.74* |
| Clu | - | - | 0.85 | 1.14 | 1.06 | 1.14 |
| Cx3cl1 | 0.90 | 1.32 | 0.86 | 0.94 | 0.70 | 1.13 |
| Cxcl16 | - | - | 0.82 | 0.85 | 0.94 | 0.73* |
| Elavl1 | - | 1.96* | 0.99 | 1.08 | 1.12 | 0.92 |
| Elp3 | 0.83 | 1.09 | 0.90 | 1.14 | 0.98 | 1.03 |
| Eml1 | 1.24 | 1.00 | 0.73* | 0.81 | 0.81 | 1.02 |
| Ep300 | - | - | 1.07 | 1.08 | 1.01 | 1.37 |
| Fbxl16 | 0.65* | 0.78 | 0.55* | 0.55* | 0.74 | 1.40 |
| Fcer1g | 0.46** | 0.77 | 0.81 | 1.18 | 0.85 | 0.91 |
| Fcgbp | 0.56** | 0.35** | 0.67* | 0.41** | 0.91 | 0.62* |
| Fcgr1 | - | - | 0.76 | 1.59 | 1.11 | 0.79 |
| Fcgr2b | 0.43** | 0.93 | 0.87 | 0.94 | 0.71* | 0.43** |
| Fcgr3 | 0.84 | 0.89 | 0.94 | 1.40 | 0.68* | 1.05 |
| Foxo1 | 2.66** | 1.36 | 1.32 | 1.40 | 2.12* | 0.89 |
| Gfap | 0.77 | 0.72 | 0.77 | 0.96 | 0.87 | 0.86 |
| Gsta1 | 1.37 | 0.89 | 0.62* | 0.87 | 0.35** | 0.67* |
| Hsf1 | 2.61** | 1.25 | 1.12 | 1.14 | 0.99 | 1.15 |
| Jakmip3 | 1.89* | 0.85 | 1.53 | 2.11** | 0.86 | 1.06 |

| Imna | 2.42** | 1.06 | 1.07 | 1.06 | 1.28 | 1.39 |
| --- | --- | --- | --- | --- | --- | --- |
| Imnb1 | 2.78** | 1.51* | 0.72 | 0.81 | 1.51* | 1.07 |
| Imnb2 | - | 1.11 | 1.72 | 1.09 | 0.98 | 0.89 |
| Ism5 | - | - | 0.70 | 0.73 | 0.84 | 0.81 |
| Ltf | - | - | 0.74 | 0.21** | 1.04 | 0.47** |
| Mbp | 0.88 | 0.96 | 0.88 | 0.87 | 1.58 | 1.01 |
| Mrpl43 | 1.57* | 2.85** | 0.97 | 1.07 | 0.88 | 1.05 |
| Ndufb11 | 1.18 | 1.28 | 0.98 | 1.19 | 1.92* | 1.27 |
| Panx1 | 1.09 | 0.43** | 0.61* | 0.80 | 0.81 | 1.21 |
| Pdcd6 | 1.72 | 1.19 | 0.90 | 1.10 | 0.81 | 0.79 |
| Phf3 | 2.52* | 1.09 | 0.82 | 1.03 | 0.79 | 0.98 |
| Polrmt | - | - | 0.91 | 1.10 | 1.24 | 1.25 |
| Pot1a | 0.86 | 0.86 | 1.32 | 1.06 | 1.28 | 0.99 |
| Rap1a | 0.99 | 0.68 | 0.79 | 0.98 | 0.89 | 1.04 |
| Rnf144b | 0.77 | 0.83 | 1.31 | 1.22 | 0.84 | 1.08 |
| S100a8 | 0.36** | 0.74* | 0.53** | 0.44** | 0.59** | 0.49** |
| S100a9 | 0.55* | 0. 84 | 0.55** | 0.41** | 0.48** | 0.54** |
| Scn2b | 0.91 | 0.77 | 0.69 | 0.79 | 0.74 | 1.04 |
| Sirt1 | - | - | 0.79 | 1.12 | 0.95 | 0.94 |
| Sirt3 | - | 0.79 | 1.15 | 1.07 | 0.76 | 0.92 |
| Sirt6 | - | - | 1.03 | 1.03 | 1.05 | 1.04 |
| Smad2 | 0.36** | 1.06 | 0.92 | 0.90 | 0.66* | 0.90 |
| Snap23 | 0.84 | 1.55 | 0.77 | 0.86 | 0.84 | 1.00 |
| Terf1 | 0.88 | 0.87 | 0.60* | 1.09 | 0.68* | 0.89 |
| Terf2 | 0.64* | 1.04 | 1.04 | 0.91 | 0.88 | 1.10 |
| Tfam | 1.15 | 1.85** | 1.24 | 1.15 | 1.73* | 0.99 |
| Tfb1m | 1.73* | 3.85** | 1.25 | 1.31 | 1.68* | 1.34 |
| Tfb2m | 1.63* | 1.82* | 1.27 | 1.20 | 1.18 | 1.17 |
| Tinf2 | 0.87 | 1.42 | 1.01 | 1.31 | 1.13 | 0.8 |
| Tlr2 | 0.44* | 1.03 | 0.57* | 0.95 | 0.77 | 0.74 |
| Tlr4 | 0.50* | 0.69* | 0.80 | 1.08 | 0.81 | 0.79 |
| Tmem135 | 0.45* | 0.99 | 0.82 | 1.01 | 0.65* | 0.86 |
| Tmem33 | - | - | 1.02 | 1.40 | 1.35 | 1.39 |
| Tollip | 0.49* | 0.82 | 0.86 | 1.13 | 0.96 | 0.89 |
| Tpp1 | 0.73 | 1.34 | 0.86 | 0.98 | 0.95 | 0.83 |
| Txnip | 0.48** | 0.85 | 0.59* | 1.15 | 0.60** | 0.73* |
| Vps13c | 0.38** | 0.80 | 0.97 | 1.04 | 0.91 | 0.96 |
| Vwa5a | 0.80 | 0.22** | 0.87 | 1.05 | 0.90 | 0.97 |
| Wrn | 0.50** | 0.95 | 0.79 | 1.07 | 0.64** | 0.76* |
| Zbtb10 | 0.45** | 0.92 | 0.74 | 0.92 | 0.78 | 0.73 |
| Zfp9 | 0.33** | 0.86 | 0.71 | 1.48 | 0.76 | 0.98 |
| Zfr | 0.99 | 0.28** | 0.81 | 1.07 | 0.85 | 0.88 |
| Zmpste24 | - | - | 1.69* | 1.13 | 0.90 | 1.75* |

Relative gene expression analysis in eWAT, iWAT, BAT, liver, muscle and brain in aging mice, control- or Rosi-treated, expressed as a fold change. n=3 for each group. Unpaired student t tests were performed to compare relative gene levels between the two groups. *, *p* <0.05; **, *p* <0.01.

**TABLE S3. Veteran Affairs Patients on Pioglitazone or Glimepiride.**

|  | N, total | N, known date of death | Median survival (days) | 95% CI, lower bound | 95% CI, upper bound |
| --- | --- | --- | --- | --- | --- |
| Glimepiride | 47987 | 10606 | 3821 | 3746 | 3873 |
| Pioglitazone | 190590 | 72022 | 4359 | 4340 | 4380 |
